# Blinding Optic Neuropathy Associated With Carboplatin Therapy: A Case Report and Literature Review

**DOI:** 10.7759/cureus.52975

**Published:** 2024-01-26

**Authors:** Sammy Shihadeh, Madison M Patrick, Galen Postma, Brenda Trokthi, Charles Maitland

**Affiliations:** 1 Clinical Sciences, Florida State University College of Medicine, Tallahassee, USA; 2 Clinical Research, Florida State University College of Medicine, Tallahassee, USA

**Keywords:** toxic optic neuropathy, optic nerve atrophy, sudden visual loss, visual field defect, optic coherence tomography

## Abstract

Various forms of cancer and chemotherapeutics are associated with optic neuropathy. Cisplatin is a platinum analogue chemotherapeutic commonly associated with ocular toxicity among many other serious adverse effects. Carboplatin is a more chemically stable platinum analogue that is generally better tolerated with a comparatively favorable side effect profile. There are very few reports of carboplatin precipitating optic neuropathy.

This case report describes a rare occurrence of carboplatin-induced blinding optic neuropathy. We treated a patient receiving carboplatin for neuroendocrine bladder cancer who developed rapidly progressive bilateral optic neuropathy over the course of three days. Upon evaluation at our clinic, his visual acuity had declined to light perception only and 20/60 in his left and right eye, respectively. Carboplatin therapy was immediately held and steroids were initiated. Despite the intervention, the patient's visual deficits have not improved at the one-year follow-up.

Although the mechanism by which carboplatin causes ocular toxicity remains speculative, arterial ischemia appears to be the likely mechanism given the irreversible nature of visual decline. As demonstrated by our patient's course, irreversible vision loss despite high-dose steroid intervention necessitates expeditious recognition and management of this rare adverse effect. ​​​​​

## Introduction

Many subtypes of cancers and chemotherapeutics are associated with optic neuropathy. The classification of such neuropathy is dependent on the mechanism of optic nerve injury, including subtypes such as infiltrative, compressive, paraneoplastic, metabolic, infectious, or ischemic [1]. Carboplatin is a commonly used platinum analogue used to treat a range of cancers. It is considered to be better tolerated than cisplatin, the other chemotherapeutic falling within this category. An abundance of current literature discusses the ocular toxicity of cisplatin and its likely ischemic mechanism of optic nerve injury [2,3]. Conversely, there are very few reports of carboplatin-induced chemotherapeutic agents causing optic neuropathy [4]. These cases discuss an assortment of visual adverse effects, including but not limited to unilateral or bilateral disc swelling, disc atrophy, and ocular or orbital inflammation. The pathogenesis of carboplatin-induced optic neuropathy remains undefined, necessitating further investigations into and reporting of this rare adverse effect. Here, we present a patient with bilateral rapidly progressive blinding optic neuropathy occurring shortly after carboplatin administration to treat bladder cancer.

This article was previously presented as an ePoster at the 2023 North American Neuro-Ophthalmology Society (NANOS) 49th Annual Meeting on March 12, 2023.

## Case presentation

A 71-year-old man with an otherwise non-contributory past medical history receiving carboplatin for the treatment of neuroendocrine-derived bladder cancer presented with acute visual vision loss over 72 hours. His precipitous visual decline began one week before after he completed the third of four total carboplatin treatment cycles. The patient's chemotherapy regimen occurred every three weeks; he received 1 mL/day of carboplatin (10 mg/mL) for three consecutive days during these treatment weeks. A total of four cycles were planned. Upon initial presentation to our clinic, the patient reported severely blurry vision with light perception in his left eye with comparatively better, albeit still distorted, vision in his right eye.

**Figure 1:**
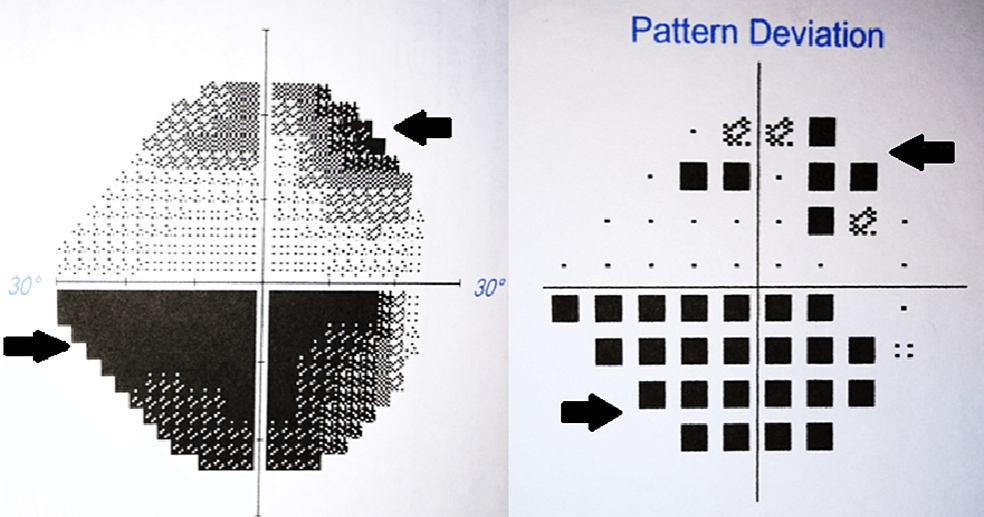
Visual field and pattern deviation mapping of the right eye four weeks post carboplatin cessation and steroid initiation An inferior altitudinal defect and superior arcuate defect of the right eye demonstrated as black regions are prominent in this figure. A visual field test was not taken of the left eye due to complete blindness.

**Figure 2:**
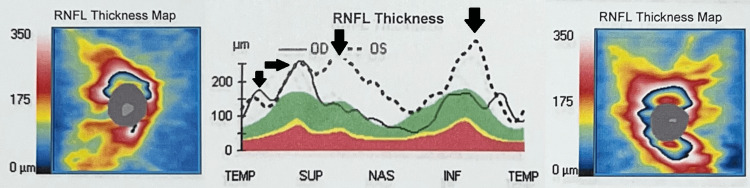
OCT of both eyes four weeks post carboplatin cessation and steroid initiation This figure demonstrates increased RNFL as conveyed by OCT, signifying optic disc swelling. OCT: optical coherence tomography; RNFL: retinal nerve fiber layer thickness

On exam, visual acuity had declined to light perception only in the left eye and 20/60 in the right eye. The left pupil was nonreactive and the right was sluggish. Extraocular movements were intact without evidence of orbital congestion. Alignment was orthophoric with full ductions and versions. Fundus examination showed bilateral grade 2 disc head swelling without hemorrhage; foveal reflexes and retinas appeared normal. No scotomas, photopsia, or metamorphopsia was present. Visual field examination revealed an inferior altitudinal defect extending through the fixation of the right eye, as shown in Figure 1. Disc head swelling was confirmed with optical coherence tomography (OCT) and is depicted in Figure 2. The neurologic exam was otherwise unremarkable.

Labs were non-contributory; complete blood count, complete metabolic panel, inflammatory markers, and coagulation studies were all normal apart from minor renal insufficiency demonstrated by an elevated creatinine. Serum titers for paraneoplastic antibodies in addition to aquaporin-4 (AQP4-IgG) and myelin oligodendrocyte glycoprotein (MOG-IgG) immunoglobulins were negative. CT and MRI of the head with and without contrast were unremarkable and without any intracranial processes. No optic nerve enhancement was seen on imaging. Subsequent lumbar puncture was unremarkable with normal opening pressure and cell counts. The temporal artery biopsy was negative.

**Figure 3:**
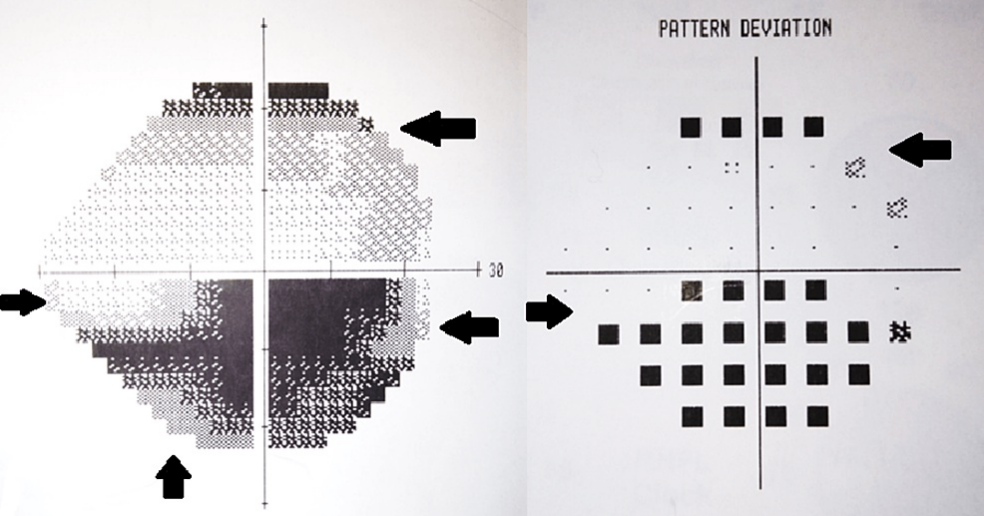
Visual field and pattern deviation mapping of the right eye eight weeks post carboplatin cessation and steroid initiation Partial recovery of the inferior altitudinal visual defect is seen here; however, the patient had not observed any meaningful changes in vision.

**Figure 4:**
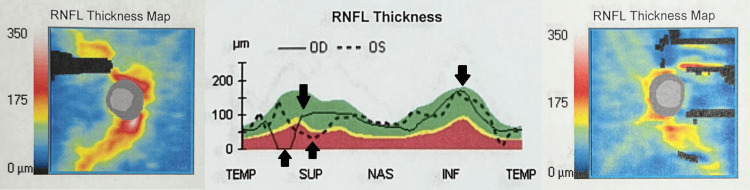
OCT of both eyes eight weeks post carboplatin cessation and steroid initiation In contrast to Figure 2, this figure demonstrates drastically decreased RNFL thickness, conveying optic nerve atrophy. OCT: optical coherence tomography; RNFL: retinal nerve fiber layer

Carboplatin treatment was immediately stopped and prednisone was initiated. The patient followed a tapered dosage schedule consisting of 80 mg daily for three weeks, followed by 60 mg daily for two weeks, 40 mg daily for two weeks, 30 mg daily for 10 days, 20 mg daily for 10 days, and finally 15 mg daily for 10 days. Despite this high-dose steroid therapy, physical re-examination eight weeks later showed minimal, partial recovery of the visual field of the right eye (Figure 3) and no improvement in visual acuity but the onset of optic atrophy in both eyes (Figure 4). This optic atrophy developed after observation of optic swelling was initially noted. Follow-up at one year showed no change in visual acuity in either eye.

Differential diagnoses

In this case, several other illnesses that might potentially cause acute visual failure were considered. Non-arteritic ischemic optic neuropathy (NAION) is common in this age group and can cause abrupt visual loss and disc swelling, sometimes bilaterally [5-7]. However, our patient lacked systemic risk factors such as hypertension and hypercholesterolemia. Further, funduscopic examination and OCT showed an open cup-disc ratio of 0.3, indicating that he did not appear to have a "disc at risk," ruling out a major risk factor for NAION, as disc at risk is suspected with a ratio of 0.2 or lower due to compression from optic disc edema [8]. Lastly, the degree of visual loss is uncommon for NAION.

Giant cell arteritis was considered, but an unremarkable temporal artery biopsy with normal levels of erythrocyte sedimentation rate (ESR) and C-reactive protein (CRP) ruled out this diagnosis. Neuromyelitis optica spectrum disorder (NMOSD) and myelin oligodendrocyte glycoprotein antibody disorder (MOGAD) were additionally considered. The more classic seropositive phenotypes were ruled out in light of the aforementioned negative antibody studies, and seronegative presentations were deemed highly unlikely given no evidence of characteristic lesion patterns on MRI. Considering a large proportion of NMOSD patients are without NMO IgG, unremarkable imaging and the lack of transverse myelitis, area postrema syndrome, or acute brainstem syndrome further ruled out a seronegative presentation. No paraneoplastic antibodies were detected. Both MRI and CT as well as lumbar puncture ruled out compressive/infiltrative processes and structural causes, such as sagittal sinus thrombosis. Idiopathic intracranial hypertension (IIH) seemed untenable given his age, sex, and normal lumbar puncture opening pressure result. Additionally, he has no IIH risk factors, and a rapidly progressive blinding optic neuropathy is not a manifestation, specifically because of how acute it was and how the patient did not have nystagmus, sixth cranial nerve (CN VI) palsy, tinnitus, or headache. 

## Discussion

It seems plausible that carboplatin toxicity caused our patient's visual failure. Carboplatin forms DNA adducts, causing inter-strand/intra-strand crosslinking that halts cellular progression; this cytotoxicity may affect the ganglion cells [9]. Carboplatin has been reported to contribute to the development of ischemic optic neuropathies. Fischer et al. documented an individual with bilateral papilledema during treatment with carboplatin [10]. Lewis et al. found evidence of unilateral optic disc head swelling after carboplatin chemotherapy for ovarian cancer [11]. The retina may also be involved; Elhusseiny and Smiddy treated a patient with ischemic retinopathy thought to be due to a combination of carboplatin and/or paclitaxel-induced ischemia [12]. Similar drugs, such as cisplatin, have also been reported to affect the anterior visual axis. Gonzalez et al. reported a patient with metastatic lung cancer who developed sudden visual loss in their left eye after a combination treatment of carboplatin and cisplatin [13]. Another patient developed bilateral central sequential visual loss 10 days after starting paclitaxel and cisplatin therapy [14]. Further details about these cases are provided below in Table 1.

**Table 1:** Additional information about other cases from the literature VA: visual acuity; RAPD: relative afferent pupillary defect; OD: oculus dexter; OS: oculus sinister; OCT: optical coherence tomography

Authors	Patient age/gender	Cancer type	Therapeutic dose	Time to symptom onset	Optical side effects	VA	Visual recovery
Fischer et al.	70/F	Serous papillary ovarian carcinoma	Thrice weekly 5 mg/ml/min for 30 minutes	Day 8 of the fourth cycle	Lack of focus/scattered blind spots in the right eye; bilateral papillary edema, particularly in the left eye; optic nerve head more prominent on the right; hemorrhage in the nerve fiber layer	Slightly reduced in both eyes; VA dropped to 20/100 in the left eye with RAPD	VA in the right eye remained stable; VA gradually recovered in the left eye over two years; optic nerve atrophy, increased pallor, and reduced visibility of the nerve fiber layer were observed in the left eye
Lewis et al.	48/F	Ovarian endometrioid carcinoma	Thrice weekly carboplatin AUC 6 (total dose 740 mg)	Five days after the fifth cycle	Blurred vision in the left eye with left optic disc edema	6/24 in the left eye; the right eye was normal with 6/6 VA	Symptoms did not improve at five months; left optic disc atrophy with 6/18 VA was observed; the right eye was asymptomatic
Elhusseiny and Smiddy	70/F	Cervical adenocarcinoma	Four cycles of paclitaxel (Taxol; Bristol Myers Squibb, New York, New York, United States) 175 mg/m^2^ and carboplatin (Paraplatin; Bristol Myers Squibb, New York, New York, United States)	Four weeks after starting chemotherapy; the previous cycle was performed six weeks prior to symptom onset	Bilateral decreased vision; free extraocular motility but normal anterior segment and pupillary reflex; peripapillary cotton wool spots more so on the left eye; OCT results were normal	20/60 OD and 20/25 OS; intraocular pressure was measured at 19 mmHg OD and 15 mmHg OS	At the 10-week follow-up, cotton wool spots resolved with unchanged VA
Gonzalez et al.	42/M	Left cerebellar adenocarcinoma with metastasis to lung	Paclitaxel 220 mg/m^2^ and carboplatin 300 mg/m^2^ over one hour, once every three weeks for six cycles; then gemcitabine 1,250 mg/m^2^ on days 1, 8, and 15; cisplatin 80 mg/m^2^ was scheduled on day 8, monthly for five months	After the fifth cisplatin/gemcitabine cycle	Severe visual loss in the left eye; discomfort started two months earlier	20/20 OD and counting fingers OS	Died one month after symptom onset due to intracranial metastasis
Li et al.	56/M	Nasopharyngeal squamous cell carcinoma	Paclitaxel 150 mg/m^2^ and cisplatin 75 mg/m^2^	14 hours after the 10th day of treatment	Blurred vision with scotoma in the left eye; central vision in the left eye was lost after 22 hours; temporal hemianopsia and nasal peripheral defects in the right eye; two days later, hypopsia in the right eye and blindness bilaterally at 72 hours	0.3 OD and light perception OS; intraocular pressures were 11 mmHg OS and 13 mmHg OD	No recovery in vision after six months

Unilateral optic neuropathy is scant in the literature, as systemic platinum analogues are observed to usually affect both sides of the optic pathway; additionally, lack of recovery of vision after cessation of the offending agent, especially despite empirical high-dose corticosteroid treatment, is also rarely documented in the literature, as most patients are shown to regain their vision. Our patient exhibiting both of these aspects demonstrates the unique, unfortunate complication that may occur with carboplatin therapy.

Furthermore, Kanat et al. illustrated the dose-dependent toxicity of platinum chemotherapeutics and conveyed that carboplatin needed a much higher dose to have similar toxicity as cisplatin and oxaliplatin (i.e., 10 times higher concentration in vitro), as well as not making reactive oxygen radicals unlike the latter drugs; in turn, this leads to cytotoxicity resulting in mitochondrial DNA damage and impairment of ion channel function [15]. This demonstrates that carboplatin has a much higher threshold for causing neurotoxicity than other platinum analogues and would therefore seem much more unusual to cause this degree of neurotoxicity.

Although the general consensus for carboplatin ocular neurotoxicity is accepted to be an immediate cessation of the chemotherapy and the administration of high-dose steroids with close monitoring, there does not appear to be a consensus on neurotoxicity prevention. A Cochrane review [16] and an American Society of Clinical Oncology panel [17] were unable to determine sufficient neuroprotective medications to prevent neurotoxicity. Thus, it is important for the patient to thoroughly understand the risks associated with the usage of carboplatin and related drugs before beginning treatment to be better informed and vigilant should any visual symptoms arise, as there is currently not a generally accepted methodology to prevent complications with vision.

## Conclusions

Carboplatin is a generally well-tolerated platinum analogue with a comparatively favorable side effect profile. There are very few reports in today's literature of it precipitating optic neuropathy or other adverse ocular manifestations. The mechanism by which carboplatin causes ocular toxicity remains speculative. Carboplatin-induced arterial ischemia seems to be the putative mechanism. However, apoptosis may be an alternative explanation. Nevertheless, carboplatin-induced optic neuropathy is not well understood. When it occurs, it may be severe and rapidly progressive and vary in presentation, as shown by our patient's pathology and symptom course, and may be irreversible despite high-dose steroid intervention. The varied unilateral or bilateral progression of carboplatin-induced optic neuropathy can delay treatment. Prodromal symptoms of vision loss should be closely monitored and necessitate expeditious recognition and management of this rare adverse effect to reduce any risk of complications and subsequent lowered quality of life.
